# Conn’s Syndrome: An Unusual Cause of Periodic Paralysis

**DOI:** 10.7759/cureus.24880

**Published:** 2022-05-10

**Authors:** Sílvia Balhana, Henrique Pina, Madalena Machete, José Maria Aragüés, Carla Noronha

**Affiliations:** 1 Internal Medicine, Hospital Beatriz Ângelo, Loures, PRT; 2 Endocrinology, Diabetes and Metabolism, Hospital Beatriz Ângelo, Loures, PRT; 3 Oncology, Hospital Beatriz Ângelo, Loures, PRT

**Keywords:** neuromuscular diseases, hypokalemia, periodic paralysis, hyperaldosteronism, conn´s syndrome

## Abstract

Primary hyperaldosteronism, also known as Conn’s syndrome, is characterized by an independent and excessive aldosterone production in the adrenal cortex, causing hypernatremia, arterial hypertension, and, in some cases, potentially severe hypokalemia can occur.

We report a case of a 45-year-old Caucasian woman, with a history of obesity and hypertension, who presented to the emergency room with a four-week evolution history of myalgia and ascending muscle weakness eventually resulting in tetraparesis. The initial blood analysis showed severe hypokalemia (1.5 mmol/L).

Further laboratory studies revealed an elevated plasma aldosterone level with low renin activity, and thyroid function tests were consistent with mild primary hyperthyroidism. CT scan showed a nodular lesion in the left adrenal gland. A saline suppression test confirmed that aldosterone secretion and renin activity were not suppressed. Regression of tetraparesis was noted with vigorous potassium supplementation. A laparoscopic left adrenalectomy was performed, with consequent normalization of hypokalemia, without the need for supplementation.

Periodic paralysis (PP) are a rare group of neuromuscular diseases that occur due to the affection of the ion channels of the skeletal muscle. Most cases are hereditary; nonetheless, secondary causes of PP have been reported in the literature.

This case illustrates an unusual and severe presentation of primary hyperaldosteronism manifested by PP. The concomitant changes in thyroid function raised the possibility that we are facing the clinical influence of another rare entity: thyrotoxic hypokalemic PP. However, the resolution of hypokalemia after the removal of adrenal adenoma supported the major contribution of hyperaldosteronism.

## Introduction

Primary hyperaldosteronism, also known as Conn’s syndrome, is characterized by an independent and excessive aldosterone production in the adrenal cortex, mostly derived from an aldosterone-producing adenoma or bilateral adrenal hyperplasia. The clinical features of primary hyperaldosteronism are mainly related to aldosterone-induced renal sodium reabsorption, causing hypernatremia, arterial hypertension, and, in some cases, a hypervolemic state. In addition, potentially severe and refractory hypokalemia can occur due to increased renal potassium excretion.

## Case presentation

A 45-year-old Caucasian woman presented to the emergency room with a four-week evolution history of myalgia and ascending muscle weakness, first noticed in the lower limbs, and then progressed to upper limbs and cervical musculature. On the day of admission, there was rapid evolution to disabling tetraparesia. She reported similar but less severe episodes of muscle weakness in the last few months, with spontaneous resolution. She also noted, in the previous three months, involuntary weight loss (10 kg), polydipsia, polyuria, and nocturia (without fever, respiratory or gastrointestinal symptoms).

The patient had a history of obesity and hypertension of young age onset (20 years) with an incomplete investigation for secondary causes because she missed follow-up appointments, but tightly controlled with an association of perindopril/amlodipine. There were no new medications, vaccines, supplements, or known toxic exposure. There were no recent travels or any relevant epidemiological contact. A high carbohydrate meal was consumed the night before the admission.

On physical examination, she was hemodynamically stable. The neurologic evaluation revealed a general diminished muscular strength with the inability to exert force against gravity. Pain, tactile, and protopathic sensitivity were intact. Deep tendon reflexes were attenuated but symmetrical in all limbs. A vesical globe was noted. A 15 mm central, well-defined, painless thyroid nodule was palpable. 

**Figure 1:**
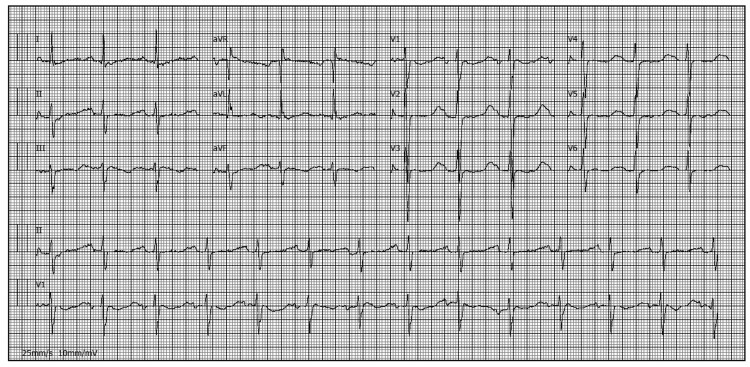
EKG showing a prolonged QT interval. EKG, electrocardiogram

The initial blood analysis showed severe hypokalemia (1.5 mmol/L, N 3,5-5,1 mmol/L), hypocalcemia (7.8 mg/dL, corrected for albumin), hypophosphatemia (1.9 mg/dL, N 2,4-5,1 mg/dL), elevated creatine phosphokinase level (12959 UI/L, N 26-192 UI/L), and elevated myoglobin (229 ug/L, N <51 ug/L). Electrocardiogram (EKG) showed prolonged QT interval with QTc of 560 ms (Figure 1).

Despite an initial period of intense potassium supplementation (in the first four hours, by central venous perfusion, with ~ 40 mEq/h), followed by maintenance of potassium infusion ~10 mEq/h, together with magnesium sulfate, for at least five days, we witnessed a very refractory ionic correction. Paradoxically, muscle strength fully recovered, with the patient being asymptomatic and able to walk on the second day of supplementation, although undercorrected potassium levels. Muscle enzyme levels decreased progressively and became normal with days. She never developed dysrhythmias or cardiovascular symptoms.

Further laboratory studies revealed an elevated plasma aldosterone level with renin activity levels being inappropriately reduced (Table 1). Thyroid function tests were consistent with mild primary hyperthyroidism. They also revealed slightly increased anti-diuretic hormone levels, probably compensatory as serum and urinary osmolality were preserved. The remaining endocrinological study was normal.

**Table 1:** Results of hormonal tests and respective reference values. The patient was under combined hormonal contraception. ADH, anti-diuretic hormone; ACTH, adrenocorticotropic hormone; DHEA, dehydroepiandrosterone-sulfate; FSH, follicle-stimulating hormone; TSH, thyroid stimulating hormone

	Patient value	Normal range
Plasma aldosterone level	22.2 ng/dL (↑)	2-9 ng/dL (in supine position and normal sodium diet)
Renin activity	0.30 ng/mL/h (↓)	0.25–5.8 ng/mL/h
ADH	8.5 pg/mL(↑)	1.0–3.6 pg/mL
ACTH	5.0 pg/mL	3.6–60.5 pg/mL
Plasma morning cortisol	8,28 ug/dL	5–25 ug/dL
DHEA	95 ug/dL	26–460 ug/dL
Delta-4-androstenedione	0.5 ng/mL	0.43–18 ng/mL
Plasma metanephrines	25 pg/mL	< 65 pg/mL
Plasma normetanephrines	107 pg/mL	< 196 pg/mL
FSH	8.1 mUI/mL	1.5–12.4 mIU/mL (for premenopausal women)
Estradiol	<19.0 pg/mL	10–30 pg/mL (for premenopausal women, receiving oral contraceptives)
Testosterone	15.7 ng/dL	15–70 ng/dL (for premenopausal women)
Prolactin	11.8 ng/mL	<25 ng/mL (for nonpregnant women)
TSH	0.07 mUI/L	0.27–4.20 mUI/L
Free thyroxine (fT4)	24,5 pmol/L	11.5–22.7 pmol/L

**Figure 2:**
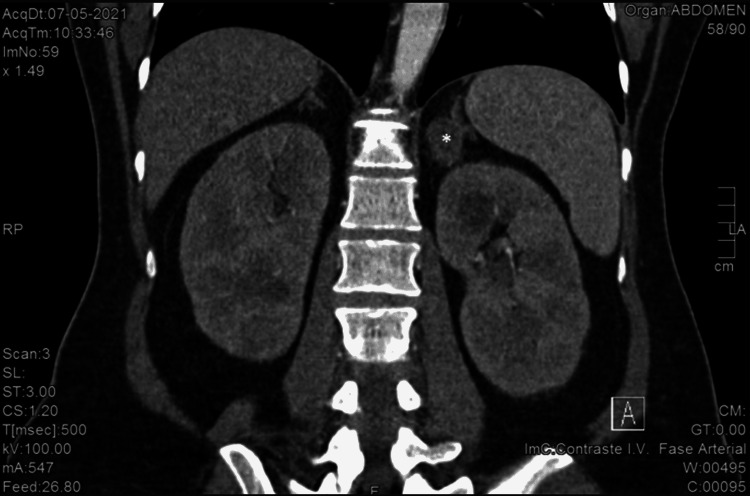
Abdominal CT showing an adenoma of the left adrenal gland (* marking the adenoma) - coronal plan.

**Figure 3:**
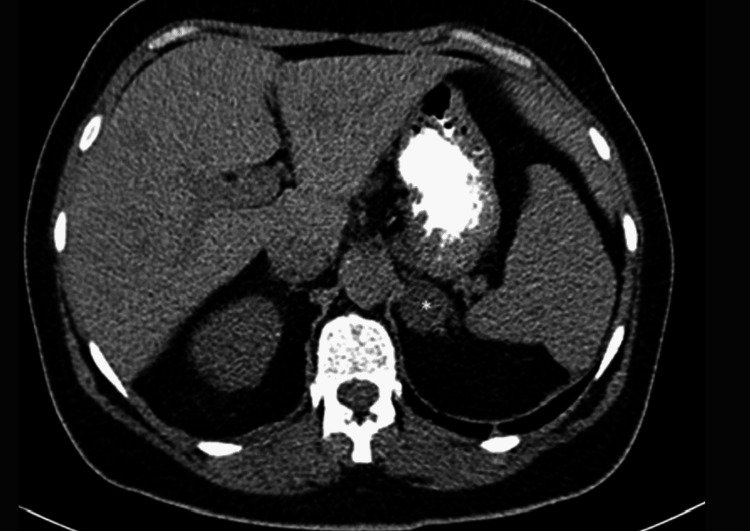
Abdominal CT showing an adenoma of the left adrenal gland (* marking the adenoma) - axial plan.

An abdominal CT was performed. It showed a 27 mm x 24 mm x 29 mm nodular lesion in the left adrenal gland, suggesting an adenoma (Figures 2-3). The right adrenal gland was normal.

Thyroid ultrasound revealed small but multiple nodules (TI-RADS 3), compatible with toxic nodular goiter after scintigraphy was performed. Anti-thyroid antibodies (anti-thyroid peroxidase - TPOAb and TSH receptor antibodies - TrAb) were negative. To exclude other causes of muscle weakness, spinal CT, cranial MRI and electromyography were performed, showing no alterations. The combination of hypertension, hypokalemia, abnormal aldosterone levels, and the presence of an adrenal mass raised the clinical suspicion of Conn’s syndrome. A saline suppression test corroborated the diagnosis as aldosterone secretion and renin activity were not suppressed after a four-hour saline infusion (Table 2).

**Table 2:** Results of saline suppression test. (*) Postinfusion plasma aldosterone levels less than 5 ng/dL make the diagnosis of primary hyperaldosteronism unlikely. Plasma aldosterone values higher than 10 ng/dL confirm primary hyperaldosteronism.

	Basal levels	After 4 h saline perfusion
Aldosterone	39,7 ng/dL (NR 2-9 ng/dL)	43,9 ng/dL (*)
Renin activity	0,20 ng/mL/h (NR 0.25–5.8 ng/mL/h)	0,20 ng/mL/h

**Figure 4:**
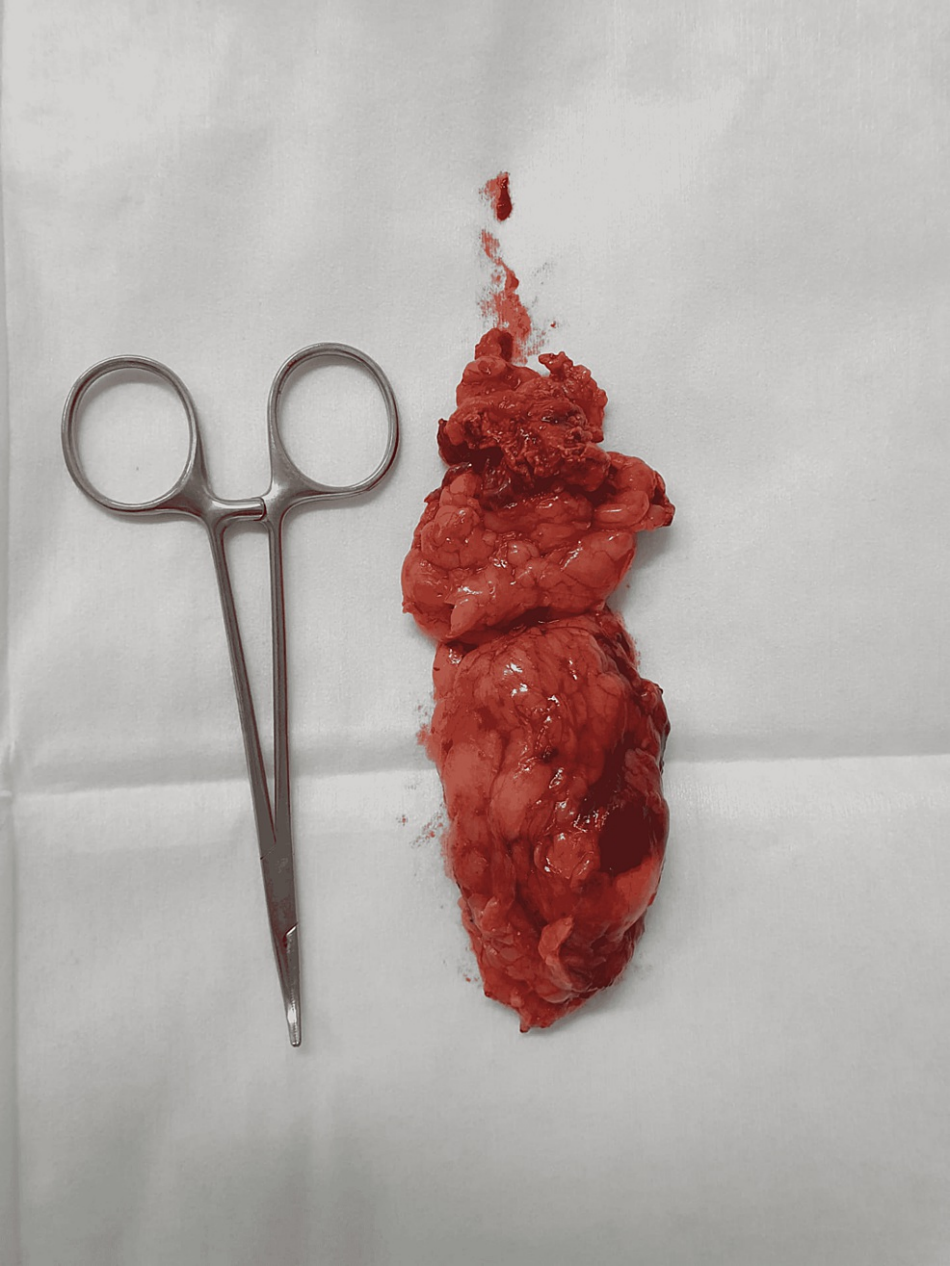
Mass removed through laparoscopic left adrenalectomy.

Treatment with spironolactone was initiated with a very good response, allowing for a progressive decrease in the daily supplemental potassium intake. After normalization of potassium levels, a laparoscopic left adrenalectomy was performed, with consequent normalization of kalemia without the need for supplementation. Histological analysis confirmed an adenoma with free surgical margins (Figure 4).

In the follow-up, three months after the surgery and without potassium supplementation or spironolactone, she was asymptomatic, without strength deficits, and with a corrected ionogram. It was possible to discontinue antihypertensive drugs, with good blood pressure control.

## Discussion

Periodic paralysis (PP) are a rare group of neuromuscular diseases that occur due to the affection of the ion channels of the skeletal muscle. Usually, they are differentiated according to blood potassium levels: hypokalemic or hyperkalemic PP [1].

They are manifested by the sudden and transient appearance of muscle weakness, usually proximal and symmetrical, starting with the lower limbs and with proximal progression. Classically, acute episodes may be preceded by a prodromal period consisting of myalgias and muscle spasms, which may be precipitated by the ingestion of carbohydrate-rich meals or after vigorous physical exercise. Other less common precipitants include trauma, exposure to cold, infection, menstruation, and the use of drugs such as diuretics, insulin, or corticosteroids. The duration of episodes may last a few minutes or several days. Most resolve spontaneously, however, severe potassium depletion may culminate in tetraplegia and death from respiratory muscle failure and/or fatal arrhythmias [1-2]. PP can also affect other systems, such as cardiovascular and gastrointestinal.

Although its exact prevalence is unknown, hypokalemic PP is estimated to affect 1 in 100,000 [2]. Most cases are hereditary, nonetheless, secondary causes of PP have been reported in the literature. Thyrotoxic hypokalemic PP is a rare but potentially fatal complication of a thyrotoxic state and it is the most frequently acquired form of secondary PP [3-4].

Primary hyperaldosteronism, also known as Conn’s syndrome, is the second most frequent endocrinopathy causing PP [5], when severe hypokalemia occurs, as in our case. This presentation is reported more frequently in East Asians and is very rare in Western countries [6].

The aldosterone-producing adrenal adenoma is the main cause of primary aldosteronism. Clinical manifestations include arterial hypertension, muscle weakness, headache, paresthesia, polydipsia, and polyuria [6]. The aldosterone to renin ratio is used to support the diagnosis (raised aldosterone levels with renin levels being normally low). Several confirmatory tests such as oral sodium loading test, saline infusion test, and furosemide upright test are used. CT imaging is helpful in detecting adrenal lesions but it carries significant false results: idiopathic adrenal hyperplasia or small adenomas (< 1 cm) can be falsely interpreted as normal in CT, and non-functioning adrenal macroadenomas can be misinterpreted as functioning tumors [7]. In certain cases, adrenal cortical scintigraphy or adrenal venous sampling performed by an experienced interventional radiologist could be helpful for differential diagnosis [8].

Surgical treatment is usually the first choice for patients with unilateral aldosterone-producing adenoma [8]. Aldosterone receptor antagonist spironolactone and potassium supplementation can be used as a bridging strategy before surgery or as an alternative option for cases not eligible for surgery.

## Conclusions

This case illustrates an unusual and severe presentation of Conn’s syndrome, with very few similar cases described in the literature in the Western population. The concomitant changes in thyroid function raised the possibility that we are facing the clinical influence of another rare entity: thyrotoxic hypokalemic PP. However, the resolution of hypokalemia after removal of the adrenal adenoma supported the major contribution of hyperaldosteronism. Early etiological identification was relevant for the institution of curative treatment and reduction of associated morbidity and mortality.
